# Protein Kinase A and C Regulate Leak Potassium Currents in Freshly Isolated Vascular Myocytes from the Aorta

**DOI:** 10.1371/journal.pone.0075077

**Published:** 2013-09-23

**Authors:** Sébastien Hayoz, Luis Cubano, Hector Maldonado, Rostislav Bychkov

**Affiliations:** 1 Department of Pharmacology and Toxicology, Michigan State University, East Lansing, Michigan, United States of America; 2 Department of Pharmacology, Universidad Central Del Caribe, Bayamon, Puerto Rico, United States of America; Leiden University Medical Center, The Netherlands

## Abstract

We tested the hypothesis that protein kinase A (PKA) inhibits K2P currents activated by protein kinase C (PKC) in freshly isolated aortic myocytes. PDBu, the PKC agonist, applied extracellularly, increased the amplitude of the K2P currents in the presence of the “cocktail” of K^+^ channel blockers. Gö 6976 significantly reduced the increase of the K2P currents by PDBu suggesting the involvement of either α or β isoenzymes of PKC. We found that forskolin, or membrane permeable cAMP, did not inhibit K2P currents activated by the PKC. However, when PKA agonists were added prior to PDBu, they produced a strong decrease in the K2P current amplitudes activated by PKC. Inhibition of PDBu-elicited K2P currents by cAMP agonists was not prevented by the treatment of vascular smooth muscle cells with PKA antagonists (H-89 and Rp-cAMPs). Zn^2+^ and Hg^2+^ inhibited K2P currents in one population of cells, produced biphasic responses in another population, and increased the amplitude of the PDBu-elicited K^+^ currents in a third population of myocytes, suggesting expression of several K2P channel types. We found that cAMP agonists inhibited biphasic responses and increase of amplitude of the PDBu-elicited K2P currents produced by Zn^2+^ and Hg^2^. 6-Bnz-cAMp produced a significantly altered pH sensitivity of PDBu-elicited K2P-currents, suggesting the inhibition of alkaline-activated K2P-currents. These results indicate that 6-Bnz-cAMP and other cAMP analogs may inhibit K2P currents through a PKA-independent mechanism. cAMP analogs may interact with unidentified proteins involved in K2P channel regulation. This novel cellular mechanism could provide insights into the interplay between PKC and PKA pathways that regulate vascular tone.

## Introduction

The aorta has a unique role in the regulation of blood pressure by adjusting to pulsatile flow. In humans, the ratio of the flow pulse amplitude to the mean flow decreases roughly from 6 in the aortic arch to less than 2 in the femoral artery. This mechanism, known as the Windkessel effect, reduces the pulse pressure, the pulse wave velocity, and the hydraulic impedance faced by the heart. Mechanical strain applied to the vascular wall alters cytosolic Ca^2+^ in myocytes of the aorta [1]. The myocyte membrane potential serves as rapid feedback that regulates Ca^2+^ concentration. Opening of the K^+^ channels hyperpolarizes the plasma membrane and inhibits Ca^2+^ influxes, while closed K^+^ channels promote the increase of the cytosolic Ca^2+^. It has been suggested that “leaky” K^+^ channels (also referred to as “background” or “baseline” K^+^ channels) or two-pore-domain K^+^ channels (K2P) lack voltage-, time-, or metabolite-dependent inactivation and thereby represent new feedback mechanisms for tuning the resting membrane potential [2], [3], [4], [5], [6], [7]. K2P channels are divided into subfamilies and are designated by acronyms such as “Tandem of P domains in weak inward rectifier K^+^ channel” (TWIK) and “TWIK-related acid-sensitive K^+^ channel” (TASK). The TASK family includes TASK-1, 2, 3, 4 and 5, although TASK-5 does not seem to produce a functional channel when expressed in artificial systems. The TWIK family comprises two members, designated TWIK-1 and 2, respectively. Other K2P subfamilies include TREK, TALK, THIK, TRAAK and TRESK channels.

Known K^+^ channel blockers do not inhibit K2P channels [8], [9], [10], [11], [12], [13]. K2P channels are regulated by a number of different G protein-coupled receptor (GPCR) pathways [14], [15]. TASK channels are inhibited following activation of the G protein Gαq, although the mechanisms are unclear [16], [17], [18], [19]. Possibly more than one pathway acts in parallel to transduce inhibition. By contrast, TRESK channels are stimulated following activation of Gαq [20]. TREK channels, the most widely regulated of the K2P channel subfamilies, are inhibited following Gαq and Gαs activation, [21], [22], [23].

We first reported that a purinergic GPCR pathway activates K2P currents in vessels [24], [25]. ATP-elicited outward K^+^ currents remained in the presence of various K^+^ channel blockers. The GPCR signaling network appeared to rely on protein kinase A (PKA) and protein kinase C (PKC) signaling molecules in the downstream activation of K2P channels. The platelet-activating factor receptor pathway signaled via PKC to inhibit TASK3 or TASK1 currents. PKC also played an inhibitory role on recombinant TASK3 channels via activation of muscarinic M3, M1 receptor and TASK1 channel via activation of the platelet-activating factor [14], [19], [26]. Knowledge of the cellular mechanisms regulating K2P channels by signaling networks employing PKA remains vague. Adrenocorticotropic hormone and cAMP may inhibit TREK-1 by a PKA-independent signaling pathway [27]. Phorbol 12,13 dibutyrate (PDBu)-induced PKC activation was shown to only partially inhibit TREK-1 channels [13], [27], suggesting involvement of other pathways in the regulation of TREK-1. It has been suggested that the agonist-induced inhibition of TREK-2 via the M3 receptor occurs primarily via PKC-mediated phosphorylation [21]. Activation of group I metabotropic glutamate receptors in heterologous expression systems inhibited TASK and TREK channels [17]. Finally, the phorbol 12-myristate-13-acetate (PMA), a specific PKC agonist, was shown to activate TRESK channels [20]. We have reported that both adenylate cyclase and phospholipase-C pathways are employed in the GPCR signaling cascades coupled to purinergic receptors in freshly isolated aortic vascular smooth muscle cells (VSMC) [24], [25], [28]. In this study, we tested the hypothesis that PKA inhibits K2P currents and investigated the interactions between PKA and PKC pathways in the aorta.

## Materials and Methods

### Ethics statement

This study was carried out in strict accordance with the recommendations in the Guide for the Care and Use of Laboratory Animals of the National Institutes of Health. The protocol was approved by the Committee on the Ethics of Animal Experiments of the University of Universidad Central del Caribe (*Permit Number: A3566-01*). Male C57BL/6 mice that were 4 weeks old were anesthetized with 2-bromo-2-chloro-1,1,1-trifluoroethane. All efforts were made to minimize suffering. Animals were purchased and housed in accordance with institutional IACUC guidelines and requirements of the relevant regulatory agencies. C57BL/6 mice were purchased from Jackson Laboratory (Bar Harbor, ME). All rodents were maintained on a 12 hr light/12 hr dark cycle with free access to food and water. Treatment, care, and housing were carried out in accordance with the National Institutes of Health guidelines on animal care.

### Smooth muscle cells dissociation and patch-clamp recording

The thoracic aorta, as previously described [28], [29], was removed, cleaned of fat tissue, and placed in a low Ca^2+^solution containing (in mM): NaCl 137, KCl 5.4, K_2_HPO_4_ 0.44, NaH_2_PO_4_ 0.42, MgCl_2_ 2, NaHCO_3_ 4.17, CaCl_2_ 0.2, glucose 11, EGTA 0.05 and HEPES 10. The pH was adjusted to 7.4 with NaOH. The aorta was incubated for 40 minutes at 37°C in low Ca^2+^ solution containing 2 mg/mL elastase (type IV) and 2 mg/mL collagenase (type II). Vascular smooth muscle cells were isolated by careful shaking of the tissue in a Ca^2+^-free solution containing (mM): NaCl 137, KCl 5.4, K_2_HPO_4_ 0.44, NaH_2_PO_4_ 0.42, NaHCO_3_ 4.17, MgCl_2_ 2, EGTA 1.8 and glucose 11 (pH was adjusted to 7.2 with NaOH) then placed on cover slips and stored at 4°C.

Membrane currents from vascular myocytes were recorded as in our previous work [30], [24], [28]. Step-pulse, linear ramps, steady state protocols and data acquisition were performed at room temperature (22–25°C). Currents were filtered at 1 kHz and digitized at 5 kHz. Membrane currents were recorded at room temperature (25°C) using a nystatin-perforated patch or whole cell configuration with an Axopatch 200 B, Axon Instruments Inc. patch amplifier. The configuration used in the experiments was as follows: The patch electrodes were pulled from borosilicate capillary glass using a Sutter instrument (P-2000, Novato, CA, USA). These had a resistance of 5–7 MΩ. Patch pipettes were filled with (mM): KCl 130, HEPES 10 (pH = 7.4). Nystatin was dissolved in DMSO and diluted into the pipette solution to give a final concentration range of 50–100 mg/mL). Recordings were delayed until full perforation of the membrane patch had been achieved, as judged from the development of repeatable currents in response to step depolarizations. This usually took 5 to 8 minutes. The aortic smooth muscle cells were bathed in a solution containing (mM): NaCl 130, KCl 5.6, MgCl_2_, CaCl_2_ 2, HEPES 8 and glucose 10 (pH 7.4). K^+^ channel blockers, PKA and PKC agonist and antagonist were added to the bath solution. The patch pipette (resistance, 4–7 MΩ) was filled with a solution containing (in mM) 80 K-aspartate, 50 KCl, 1 MgCl2, 3 Mg-ATP, 0.5 EGTA, 5 K-HEPES (pH 7.4). The same solution was used in perforated and whole-cell patch clamp experiments. In a sample of 20 cells the average cell capacitance was 18±4 pF and the series resistance was ≤12 MΩ in whole-cell configuration and ≤23 MΩ in perforated patch clamp configuration. The “cocktail” of K^+^ channel blockers used to inhibit conventional K^+^ channels contained: TEA (3 mM), 4-AP (3 mM), apamin (1 µM), charybdotoxin (200 nM) and glibenclamide (10 µM). The linear voltage ramps were applied from a holding potential of −60 mV for 500 ms duration and at voltages ranging from −100 to 100 mV. To elicit whole-cell currents and to build current-voltage relations 300 ms voltage steps were applied from the holding potential of −60 mV with 10 mV increments from −100 to 100 mV. Current densities (pA/pF) were obtained for each cell by normalization of whole cell current to cell capacitance to account for differences in cell membrane surface area. Capacity currents were measured for each cell during 10-ms pulses from a holding potential of −80 mV to a test potential of −70 mV. Currents were digitized and recorded at 5 kHz without filtering. Cell capacitance was calculated from the capacitive current transient recorded at the beginning of a 10 mV depolarizing voltage-clamp pulse. The total charge movement (Q) during the 10 mV step was obtained by integrating the area defined by the capacitive transient. Cell capacitance (C) was then obtained from the equation C = Q/V.

Currents elicited by a voltage ramp were used to build up dose response curves of the inhibition of K^+^ currents by Hg^2+^ and Zn^2+^. The area under the curve (AUC) of the current/voltage relationships elicited by linear voltage ramps from −100 to 100 mV was calculated. The AUC was normalized to the membrane capacitance to avoid variations of the current due VSMC size and was used to construct dose-response curves. Means of the dose response data sets were fitted using a logistic equation: Y = (A1−A2)/(1+(x/k)n))+A1; where A1 is the normalized maximum, A2 is the maximum inhibition, k is the EC50 concentration, and n is the slope factor. The error associated with these values represents the error in the fit to the mean data. P values of ≤0.05 were considered to be significant.

### Drugs

Iberiotoxin, charybdotoxin, TEA, 4-aminopyridine (4 AP), glibenclamide, apamin, ethylene glycol-bis (2-aminoethylether)-*N*,*N*,*N*′,*N*′-tetraacetic acid (EGTA), elastase (type IV from porcine pancreas), 6-benzoyl-cAMP (6-Bnz-cAMP), *N*-[2-(4-bromocinnamylamino)ethyl]-5-isoquinoline (H-89), adenosine 3-5-cyclic-monophosphothiate (Rp-isomer), Phorbol 12,13 dibutyrate (PDBu), Gö 6976, zinc chloride and mercury chloride were obtained from Sigma Chemical (USA).

### Data analysis and statistics

Patch-clamp data were processed using Clampfit 9.0 (Molecular Devices) and then analyzed in Origin 7 (Origin Lab, Northampton, MA, USA). Numerical data in the text and error bars in the figures are expressed as mean ± S.E.M. Whenever possible, differences in I–V plots and dose response relationships were analyzed using two-way analysis of variance (ANOVA). Otherwise, data were analyzed by t-tests. P values of ≤0.05 were considered to be significant.

## Results

### Intracellular cAMP does not inhibit PDBu elicited K2P currents

**Figure 1:**
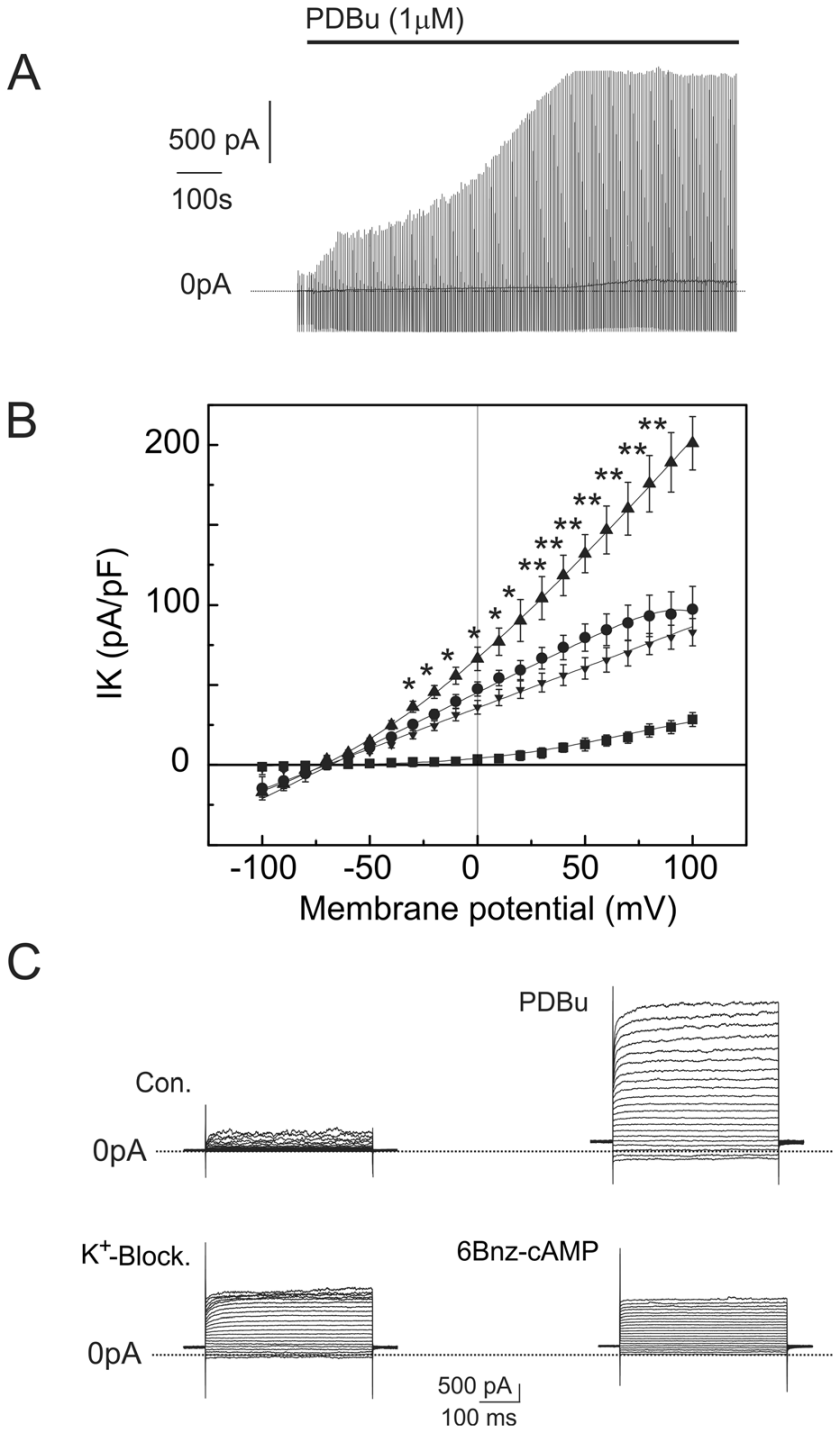
PDBu (1 µM) increases the amplitude of outward K^+^ currents. Panel A: K^+^ currents elicited by linear voltage ramps varying from −100 to 100 mV. Data are displayed in a concatenated pattern. Application of PDBu to the superfusing solution is indicated by a bar line. Panel B: current-voltage relationship recorded in the control (squares); after application of the PKC agonist PDBu (up triangles); after application of the “cocktail” of K^+^-channel blockers (circles); and after application of the membrane permeable cAMP analog 6-Bnz-cAMP (300 µM) (down-triangles). K^+^ channel blockers significantly inhibited a fraction of the PDBu-elicited K^+^ current (*P<0.01, **P<0.001, by two-way ANOVA). 6-Bnz-cAMP did not significantly decrease the amplitude of the PDBu-elicited currents (p>0.05, by two-way ANOVA). Panel C: example of the superimposed families of the currents used to build up the current-voltage relationships. Currents were elicited by voltage steps from −100 mV to 100 mV with the increment of 10 mV from holding potential of −60 mV. K^+^ currents were recorded in control (Con.); after application of the PKC agonist PDBu (PDBu); after application of the “cocktail” of K^+^ channel blockers (K+-Block.); and after application of the membrane permeable cAMP analog 6-Bnz-cAMP (6-Bnz-cAMP).

PDBu (1 µM) typically increased the amplitude of the outward K^+^ currents recorded in both perforated patch-clamp and whole cell configurations. The onset of the PDBu-elicited K^+^ currents was 15.6±2.1 and 10.7±1.4 minutes respectively (t-test, p<0.05; n = 16). The cocktail of K^+^ channel blockers applied to the solution partially inhibited PDBu-elicited K^+^ currents (Fig. 1, panel B and C). Outward PDBu-elicited K^+^ currents shared properties with leak K^+^ currents, identified as two-pore domain K^+^ currents (K2P currents) in our previous reports [24], [25].

Vascular smooth muscle expresses several PKC isoenzymes—usually α, β, δ, ε, and ζ are present [31]. We tested the effect of the selective inhibitor of conventional PKC isoenzymes, Gö 6976. Cells were pretreated with Gö 6976 (1 µM) for one hour and PKC inhibitor was present in the extracellular solution throughout the experiment. Gö 6976 significantly reduced the increase of the K2P currents by PDBu to 24.0±2.8% (n = 5, t-test P<0.01) vs. the control, suggesting the involvement of either α or β isoenzymes of PKC.

It has been suggested that protein kinase A (PKA) and cAMP decrease the open probability of several types of the K2P channels [27], [32], [33]. We therefore tested the hypothesis that PKC activates and PKA inhibits K2P currents in aortic VSMC. cAMP analogs modified at the 6 positions of the adenine ring have been found to bind to PKA. In vitro, membrane permeable 6-Bnz-cAMP activates PKA at submicromolar concentrations [34], [35]. However, in perforated patch-clamp recordings, external application of 6-Bnz-cAMP (300 µM) for 15–20 minutes in the presence of K^+^ channel blockers failed to reduce the amplitude of the PDBu-elicited current (Fig. 1, panel B and C). The failure of 6-Bnz-cAMP (300 µM) to inhibit K2P currents may indicate that either this cAMP derivative did not reach the intracellular concentration necessary to activate PKA, or that PKA has no effect on K2P currents in the aorta. The last hypothesis is supported by the data showing that protein kinases may oppositely affect the same type of ion channels in aorta and small arteries. Protein kinase C, for example, was reported to inhibit delayed rectifier and Ca^2+^-dependent K^+^ currents in vascular smooth muscle cells (VSMCs) of small muscular arteries [36], [37], [38]. Whereas, PKC activated by PDBu (1 µM) increases amplitude of the outward K^+^ currents in VSMCs freshly isolated from the aorta (Fig. 1, Panel B and C) and cocktail of conventional K^+^ channel blockers fractionally inhibited the outward K^+^- current. Unique structure and function [39], [40], [41] places the aorta apart from other blood vessels and allows speculation that this type of opposed regulation of ion channels amplifies the vasodilation needed for the maintaining of Windkessel mechanism of the aorta with changes of the blood pressure.

**Figure 2:**
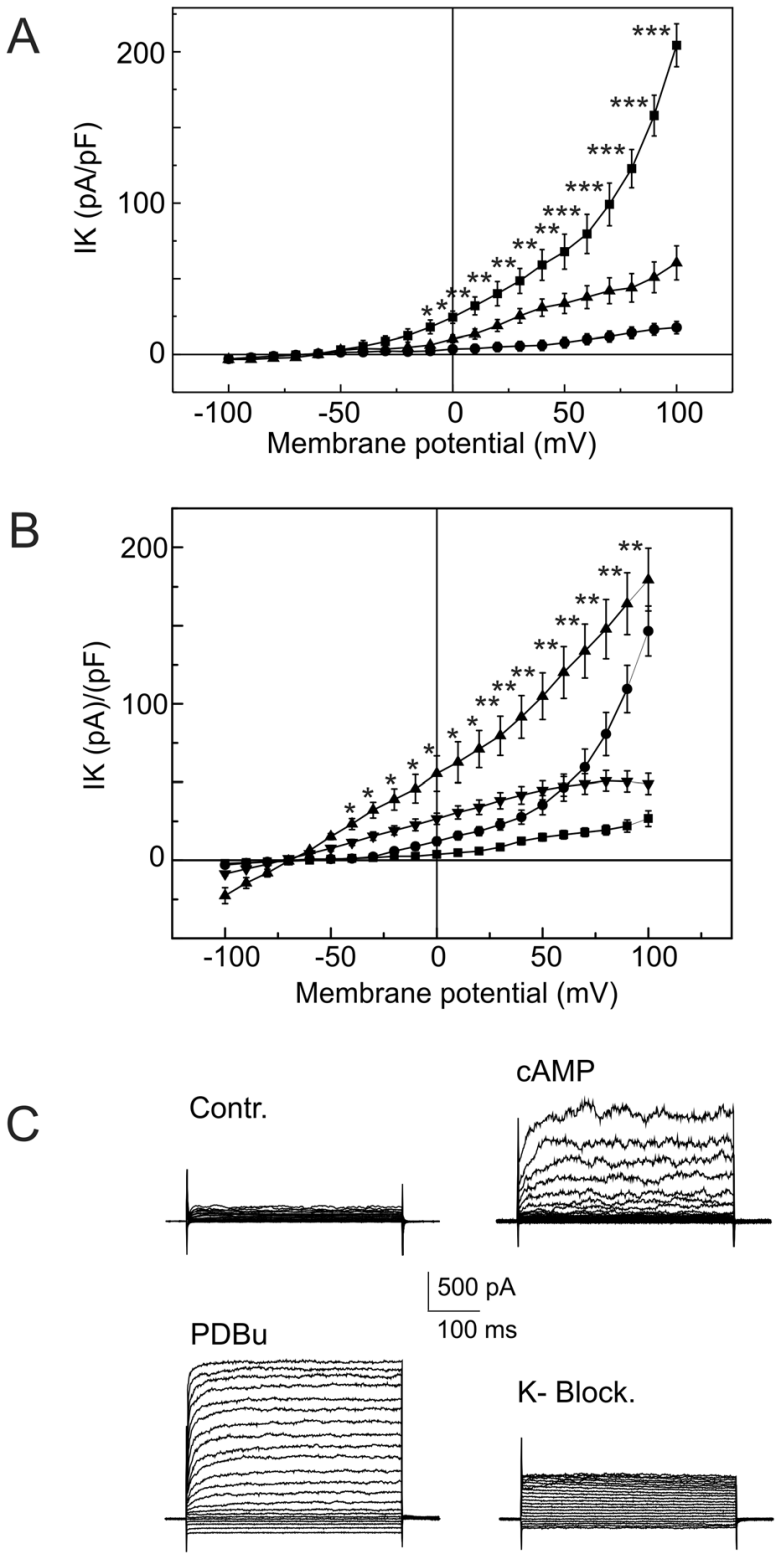
Action of cAMP agonists on PDBu-elicited K^+^ currents recorded in freshly isolated myocytes from the mouse aorta. Panel A: Current-voltage relationship recorded in control (up triangles); after application of forskolin (1 µM) (filled squares); and after application of the “cocktail” of K^+^ channel blockers (circles). Forskolin significantly increased the amplitude of the K^+^ currents (*P<0.05, **P<0.01, ***P<0.001 by two-way ANOVA). K^+^ channel blockers significantly inhibited the forskolin-elicited K^+^ current (P<0.001, by two-way ANOVA). Panel B: Current voltage relations recorded in the control (squares); after application of the membrane permeable cAMP analog 6-Bnz-cAMP (300 µM) (circles); after application of the PKC agonist PDBu (up triangles); and after application of the “cocktail” of K^+^ channel blockers (down-triangles). The cocktail of K^+^ channel blockers significantly inhibited the 6-Bnz-cAMP-elicited K^+^ current (*P<0.01, **P<0.001, by two-way ANOVA). Panel C: example of the superimposed families of the currents used to build up the current-voltage relationships. Currents were elicited by voltage steps from −100 mV to 100 mV in increments of 10 mV from holding potential of −60 mV. K^+^ currents were recorded in the control (Contr.); after application of the membrane permeable cAMP analog 6-Bnz-cAMP (cAMP); after application of the PKC agonist PDBu (PDBu); and after application of the cocktail of K^+^ channel blockers (K+-Block.).

We reported earlier that forskolin activated BK_Ca_ currents in aortic VSMC by increasing intracellular cAMP and PKA activation [28]. Forskolin (1 µM) used as an alternative to 6-Bnz-cAMP was found to not inhibit PDBu-elicited K2P currents (n = 7) in the presence of K^+^ channel blockers (data not shown). Forskolin was also applied to the bath solution prior to PDBu to test the hypothesis that the cAMP/PKA pathway may activate K2P currents alone in parallel with the PKC pathway (Fig. 2, panel A). Myocytes were exposed to forskolin for 15–30 minutes. Forskolin did increase the amplitude of the outward K^+^ currents. However, the current/voltage relationships and pharmacological properties of the forskolin-elicited currents were different from PDBu-elicited K^+^ currents. The current/voltage relationship showed strong voltage dependence with outward rectification, in contrast to current/voltage relationships recorded in the presence of PDBu. The K^+^ channel blockers completely inhibited forskolin-elicited K^+^ currents (n = 5), whereas the same “cocktail” only partially inhibited PDBu-elicited currents.

### PDBu activates fewer K2P currents in myocytes loaded with cAMP agonist

PKA and PKC may phosphorylate separated sites on the K2P channels [42], [15]. It has also been suggested that PKA phosphorylates TREK1 and converts it into a voltage-dependent channel [43]. We hypothesized that PKA may change the gating of K2P channels and thereby modify activation of K2P currents by PKC. To test this idea, myocytes were superfused for 15–20 minutes with 6-Bnz-cAMP (300 µM) (n = 7) or forskolin (1 µM) (n = 9) before PDBu application. Both agonists increased the outward K^+^ currents amplitude. Current/voltage relationships showed strong voltage dependence with exponential growth. The activation threshold was shifted to more negative potentials from −9.8±1.6 mV to −27.9±2.8 mV (t-test p<0.001) by 6-Bnz-cAMP and from −11.2±1.4 mV to −30.8±2.4 mV (t-test p<0.001) by forskolin.

PDBu (1 µM) increase the net outward K^+^ currents additively to 6-Bnz-cAMP and forskolin. The cocktail of K^+^ channel blockers inhibited a fraction of the K^+^ currents stimulated by 6-Bnz-cAMP. Current voltage relationships and families of K^+^ currents, evoked by step pulses in control and after application of 6-Bnz-cAMP and PDBu, are shown in Figure 2. The effects of forskolin were similar to the 6-Bnz-cAMP K^+^ currents evoked by PDBu superfusion. First, forskolin increased the K^+^ currents amplitude from 19.4±3.2 pA/pF to 52.1±3.8 pA/pF, measured at 50 mV (t-test p<0.001). PDBu further increased the K^+^ currents amplitude to 120.4±7.8 pA/pF, measured at 50 mV (t-test p<0.001) and the cocktail of K^+^ channels blockers inhibited a fraction of stimulated currents to 67.4±4.2 pA/pF (t-test p<0.001). To further strengthen these results, 6-Bnz-cAMP (n = 4) and forskolin (n = 5) were added to the pipette solution in separate experiments to dialyze VSMC after membrane rupture in the patch. We did not find differences in responses to PDBu between VSMC super-fused or dialyzed with 6-Bnz-cAMP or forskolin.

**Figure 3:**
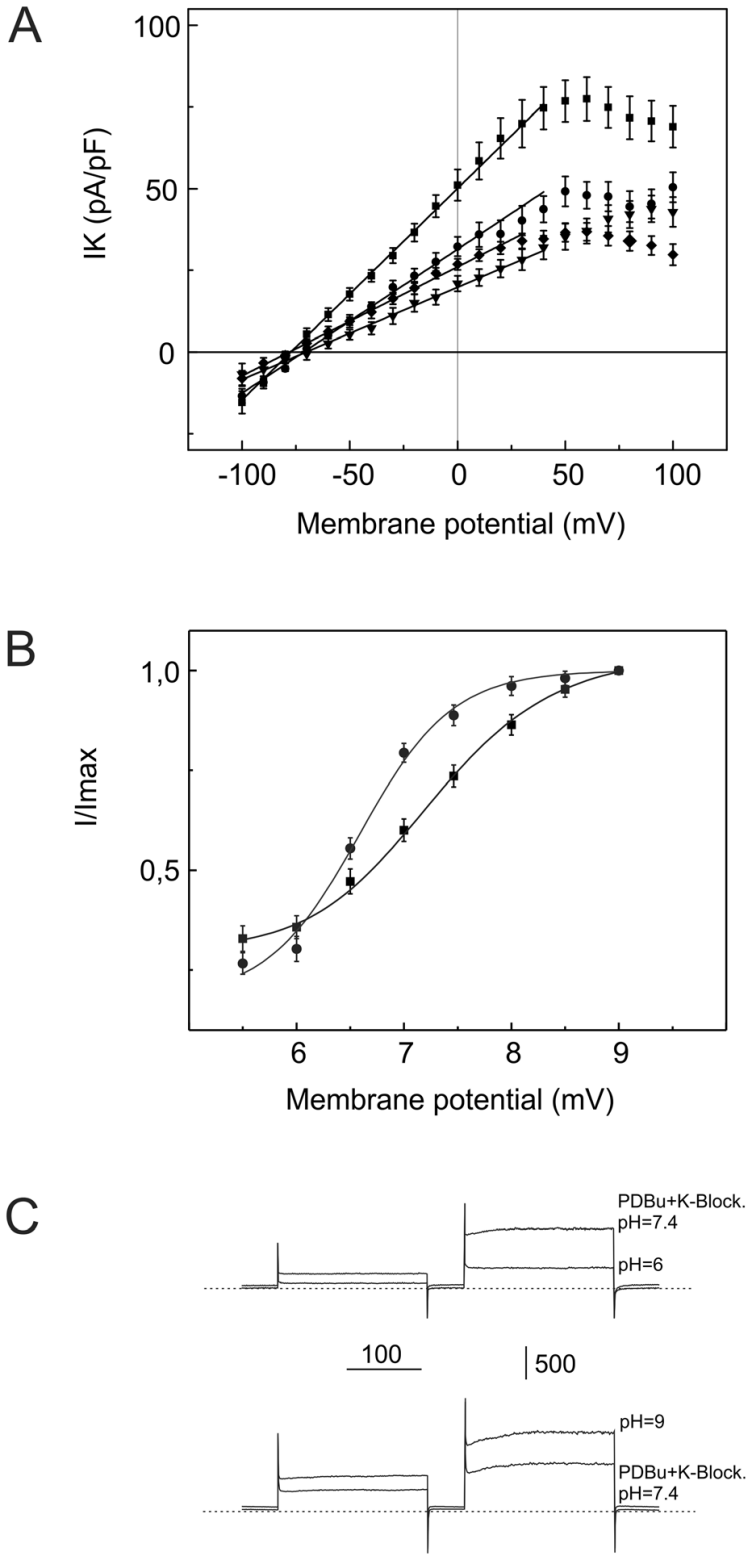
Comparison of the current-voltage relationships obtained by subtraction of the control currents from PDBu-elicited K^+^ currents with the cocktail of K^+^-channel blockers for four experimental conditions (Panel A). 1) 6-Bnz-cAMP (300 µM) was added to the bath solution after the PKC agonist PDBu (squares). 2) Cells were pretreated with 6-Bnz-cAMP (300 µM) for for 15–20 minutes before application of PDBu (diamonds). The two other current voltage relationships show PDBu-elicited K^+^ currents in the presence of cocktail of K^+^ channel blockers recorded from cells pretreated with PKA antagonist H-89 (1 µM). 3) Forskolin (1 µM) was applied to the bath solution before PDBu (circles). 4) Cells were dialyzed with 6-Bnz-cAMP (30 µM) and Rp-cAMPS (300 µM). 6-Bnz-cAMP (300 µM) was also added to the bath solution before application of PKC agonist PDBu (down-triangles). cAMP agonists inhibited significantly PDBu-elicited K^+^ current (P<0.01, by t-test). Panel B. Effects of extracellular pH on the PDBu-elicited K^+^ currents recorded in freshly isolated myocytes from the mouse aorta. Currents were recorded at different pH values and were normalized to membrane capacitance and to the maximum at pH 9. Response curves for control cells (squares) and for cells pretreated with 6-Bn-cAMP (circles). PDBu-elicited K^+^ currents with changes in extracellular pH are shown. 6-Bnz-cAMP significantly shifted the response curve to the low pH values (p<0.01 t-test). Panel C shows representative PDBu-elicited K^+^ currents, as well as the effect of pH = 6 (upper trace) and the effect of pH = 9 lower trace. Currents were elicited by step pulses to 0 mV and to 50 mV from holding potential of −60 mV.

PDBu elicited K2P currents recorded in the presence of K^+^ channel blockers in VSMCs pretreated with forskolin or with 6-Bnz-cAMP, or dialyzed through pipette solution, varied virtually linearly with voltage from −100 to 30 mV, similar to what was found for the K2P current voltage dependence in the control VSMC. The noticeable difference was that the amplitude of the K2P currents activated by PDBu in VSMC pretreated with cAMP agonists was significantly smaller than the amplitude of K2P currents recorded in the control cells (Fig. 3, Panel A).

### PKA antagonists do not block cAMP-mediated K2P currents inhibition

In light of the finding that increased intracellular cAMP inhibited a large fraction of the whole cell K2P channel currents stimulated by PKC, we next tested whether or not cAMP inhibited K2P currents by PKA dependent mechanisms. We used the potent membrane-permeable PKA antagonist, H-89 [44], [45]. Fresh aortic VSMC were pre-incubated with H-89 (1 µM) for 10–15 minutes before whole cell K^+^ currents were recorded. The current/voltage relationships of the K^+^ currents recorded in VSMC pretreated with H-89 were similar to current voltage relations recorded in control cells. 6-Bnz-cAMP was added to the bath solution (300 µM) or to the pipette solution (10 µM) prior to the application of PDBu. H-89 failed to prevent inhibition of the PDBu-elicited K2P currents by intracellular cAMP (Fig. 3, panel A). Thus, under conditions in which PKA activation was abolished by H-89, 6-Bnz-cAMP added to the bath (n = 7), or to the pipette solution (n = 5), as well as forskolin (n = 7), continued to inhibit part of the PDBu stimulated K2P currents. As an alternative to H-89, we used Rp-cAMPS that inhibits activation of PKA by endogenous cAMP due to competitive binding to PKA [46], [35]. In the next series of experiments myocytes were dialyzed with Rp-cAMPs (300 µM) together with 6-Bnz-cAMP (30 µM) and cells were superfused with H-89 (1 µM). Rp-cAMPs did not prevent cAMP-mediated inhibition of the PDBu-elicited K2P currents under these experimental conditions (n = 4) (Fig. 3, Panel A).

### Effects of pH on PDBu-elicited K2P currents

A hallmark of K2P channels is their sensitivity to pH. Among K2P channel subunits, 10 are sensitive to variations of the extracellular pH value. To determine whether PDBu-elicited currents possess similar pH sensitivity in control cells and in cells pretreated with cAMP analogs, we examined pH dependent responses. PDBu-elicited currents were measured at different pH values (Fig. 3, Panels B and C). PDBu-elicited current was markedly inhibited by extracellular acidification (pH 5.5–7.0) at all membrane potentials in both control cells and in cells pretreated with 6-Bnz-cAMp (300 µM). An increase in pH above 7.4 caused a rise in PDBu-elicited current, showing that this current is also sensitive to changes in pH in the alkaline range (8.0–9.0). 6-Bnz-cAMp produced a significant shift of the pH sensitivity of PDBu-elicited currents. Averaged currents at different pH values were measured from 4–7 different cells and plotted against the corresponding pH. Amplitudes of the PDBu-elicited current were normalized to the maximum amplitude recorded at alkaline pH = 9. The pH under which PDBu-elicited current had 50% of the maximal amplitude was 7.2±0.08 for control VSMCs and 6.5±0.04 for VSMCs pretreated with 6-Bnz-cAMP. The Hill coefficient of the fitted curve was 0.9±0.08 for control and 1.0±0.07 for 6-Bnz-cAMP pretreated VSMCs.

### Effects of zinc and mercury on PDBu-elicited K2P currents

To date, there is a lack of selective pharmacology for K2P channels. Among available pharmacological tools, Zn^2+^ and Hg^2+^ have been widely used in expression systems and in neuronal tissue as specific modulators of K2P channels [47], [48], [13], [49]. It is generally agreed that in expression systems Zn^2+^ and Hg^2+^ enhance the amplitude of TREK1 and TREK2 and strongly decrease the amplitude of the rodent TRESK and TASK3 currents. The intensity of the effect of Zn^2+^ on TASK1, TASK2 and TASK3 channels seems to depend on the expression system. Furthermore, species differences of the effects of divalent cataions on K2P channels have been reported.

**Figure 4:**
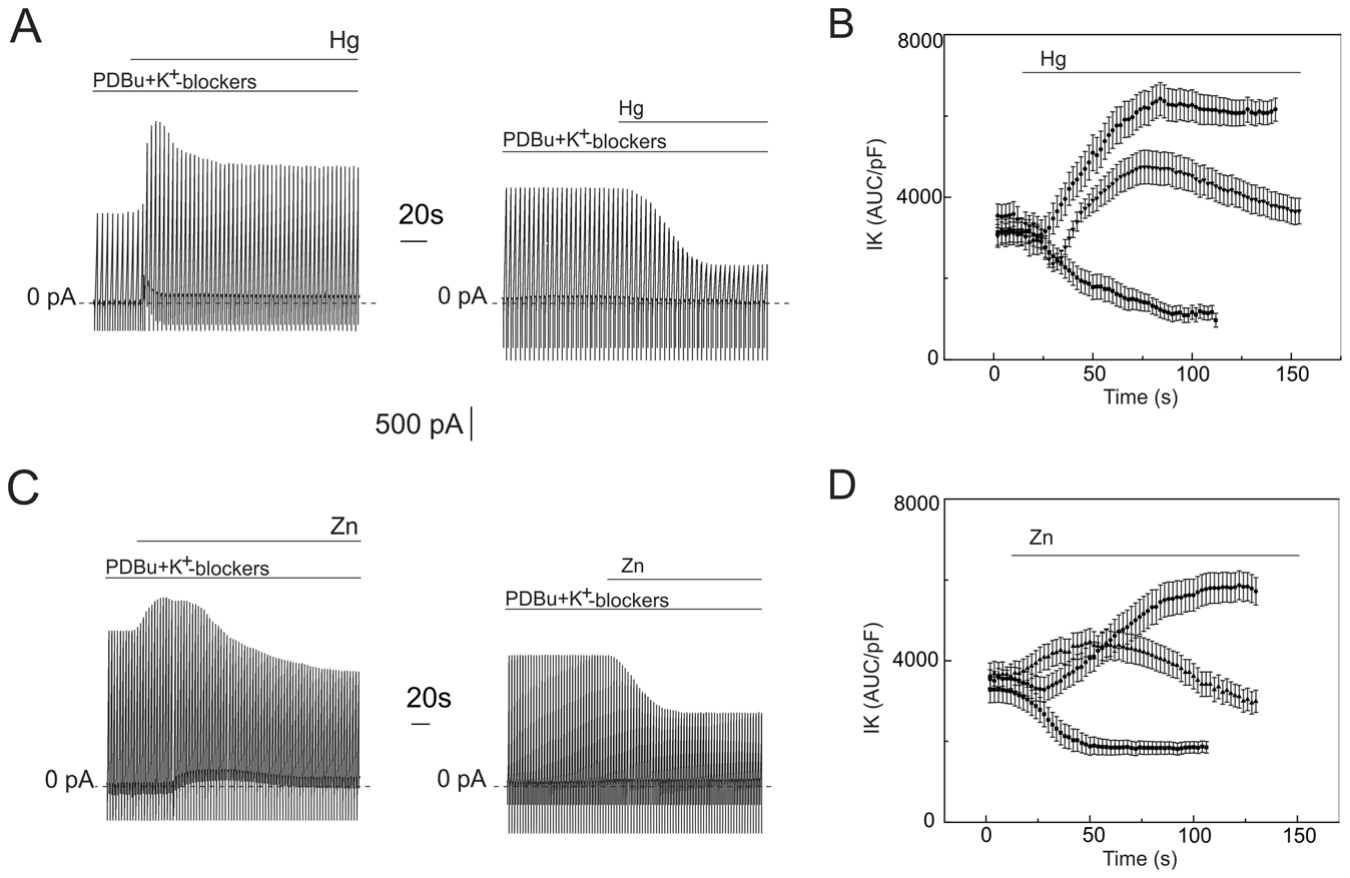
Effects of mercury and zinc on PDBU-elicited K^+^ currents. Outward K^+^ currents were elicited by linear voltage ramps varying from −100 to 100 mV. Data are displayed in concatenated pattern. K^+^ currents are shown in the presence of PDBu (1 µM) and the cocktail of K^+^ channel blockers. Application of mercury (10 µM) and zinc (50 µM) is shown by the horizontal line bar above each trace. Panel B and D: Currents recorded from five VSMC representing each type of response were analyzed. The area under curve (AUC) calculated from the K^+^ currents elicited by linear voltage ramps (examples shown in Panel A and C) were normalized to the membrane capacitance (pF) and plotted against time. To illustrate biphasic response VSMC with pronounced two phases were especially chosen.

According to previous reports, the concentration of Zn^2+^ (50 µM) and Hg^2+^ (10 µM) used in our experiments might fully inhibit one type, and at least partially enhance another type of K2P currents. The amplitude of the PDBu-elicited K2P currents was measured at 50 mV to analyze the effect of divalent cations. PDBu-elicited currents showed three types of responses to Zn^2+^ and Hg^2+^ in the presence of a cocktail of K^+^ channel blockers. Zn^2+^ (50 µM) and Hg^2+^ (10 µM) decreased PDBu-elicited K2P currents in the first population of VSMCs by 54.8±6.2% (t-test p<0.001, n = 9) and by 65.8±5.1% (t-test p<0.001, n = 12) respectively (Fig. 4, Panel A and C right traces). In the next group of VSMCs, Zn^2+^ (n = 8) and Hg^2+^ (n = 10) evoked bi-phasic types of responses, suggesting that PKC activated by PDBu may affect several types of K2P channels simultaneously (Fig. 4, Panel A and C left traces). The amplitude and duration of the phases may depend on the types of K2P channel expressed in particular cells and on the binding affinity of divalent cations to K2P channels. This group of cells was not used in the analysis because it was difficult to interpret what type of K2P channel was affected by Zn^2+^ and Hg^2+^. Finally, Zn^2+^ and Hg^2+^ increased the amplitude of the PDBu-elicited K2P current by 43.8±6.4% (t-test p<0.01, n = 8) and by 78.2±9.2% (t-test p<0.001, n = 9) respectively.

We tested the hypothesis that cAMP agonists may also affect the type of response of the PDBu-activated K2P currents produced by Zn^2+^ and Hg^2+^. Myocytes were pretreated with 6-Bnz-cAMP (300 µM) or with forskolin (1 µM) for 10–15 minutes before PDBu application. Both tested cAMP agonists produced similar results. Zn^2+^ (50 µM) and Hg^2+^ (10 µM) inhibited PDBu-elicited currents like in control cells by 46.2±3.2% (t-test p<0.001, n = 9) and by 74.8±2.1% in 6-Bnz-cAMP pretreated cells (t-test p<0.001, n = 12) respectively. However, cAMP agonists inhibited the increase of amplitude and biphasic response of the PDBu-elicited currents induced by Zn^2+^ and Hg^2+^.

**Figure 5:**
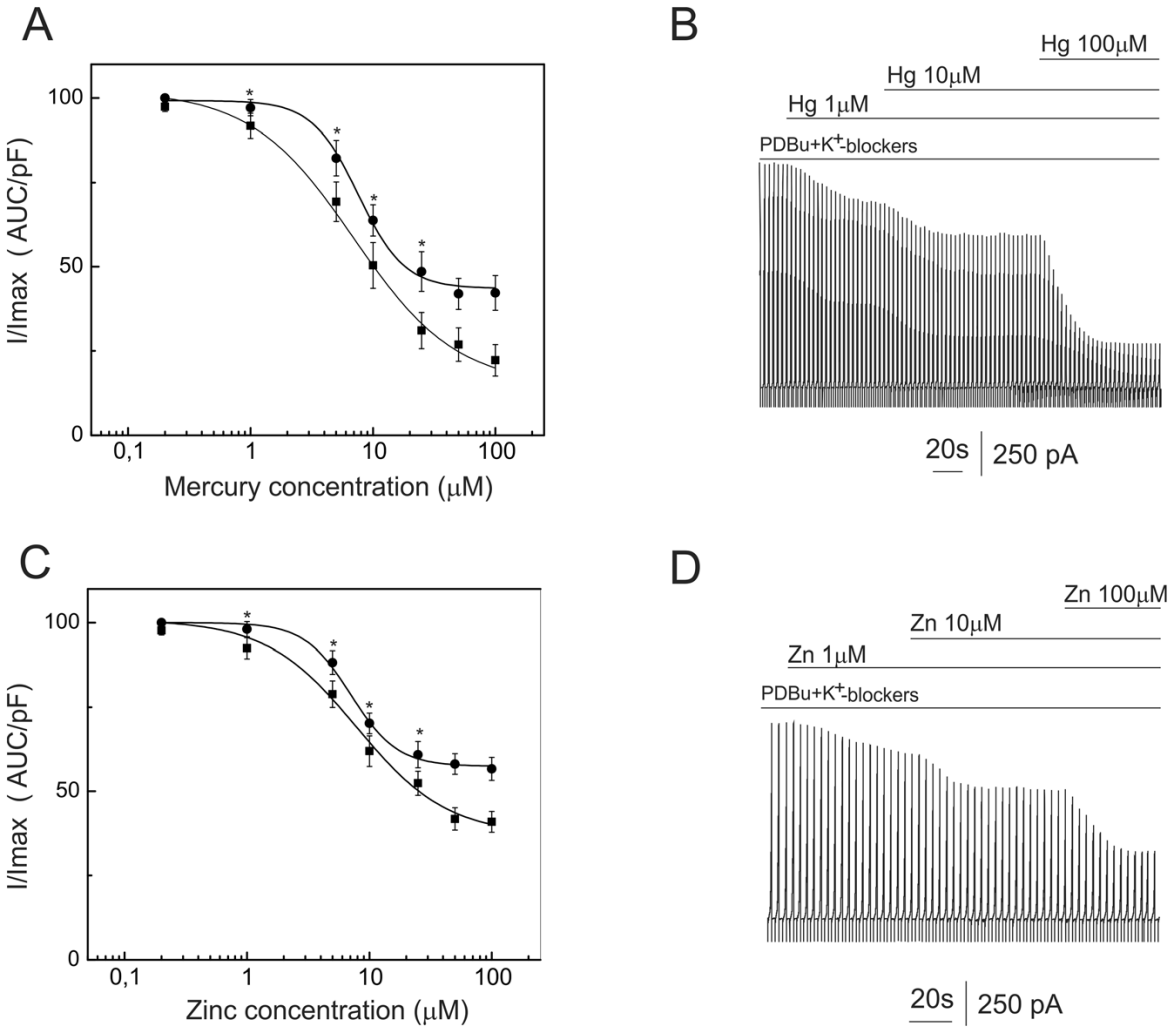
Dose response inhibition of PDBu-elicited currents produced by zinc and mercury recorded in the presence of the “cocktail” of K^+^-channel blockers. K^+^ currents were elicited by linear voltage ramps varying from −100 to 100 mV. The area under curve (AUC) was calculated and normalized to membrane capacitance (pF), following which the data sets were normalized to the maximum value. Panel A: dose response curve of the inhibition of PDBu-elicited K^+^ current by mercury was fitted with logistic function. PDBu-elicited K^+^ currents were recorded in control cell (circles) and in cells pretreated with 6-Bnz-cAMp (300 µM) (squares). EC50 calculated for control cells was 7.6±1.2 µM and the slope was 2.3±0.3. EC50 calculated for cells pretreated with 6-Bnz-cAMP was 6.9±1.5 µM and the slope was 1.1±0.2. Panel B: dose response curve of the inhibition of PDBu-elicited K^+^ current by zinc was fitted with logistic function. PDBu-elicited K^+^ currents were recorded in control cell (circles) and in cells pretreated with 6-Bnz-cAMp (300 µM) (squares). EC50 calculated for control cells was 7.4±0.2 µM and the slope was 2.2±0.1. EC50 calculated for cells pretreated with 6-Bnz-cAMP was 7.9±1.4 µM and the slope was 1.2±0.2. Panels C and D: examples of PDBu-elicited K^+^ currents with the cocktail of K^+^ channel blockers in the bath solution. K^+^ currents were elicited by linear voltage ramps varying from −100 to 100 mV. Data are displayed in a concatenated pattern. The application of mercury (Hg) and zinc (Zn) is shown by the horizontal line bars above the panels. 6-Bnz-cAMP did not significantly alter EC50 of the Zn^2+^ and Hg^2+^ inhibited currents (P>0.05, by t-test).

Figure 5 shows the concentration response curves for Zn^2+^ and Hg^2+^ inhibition of PDBu-elicited currents in the control and in VSMC pretreated by 6-Bnz-cAMp (300 µM). In these cases, superfusion of VSMCs with extracellular solution was not done, and Zn^2+^ and Hg^2+^ (from prepared stock solutions) were applied cumulatively to the cocktail of K^+^ channel blockers. Stock solutions contained PDBu, a cocktail of K^+^ channel blockers, and 1 mM or 10 mM of the divalent cations. A maximum of three concentrations of Zn^2+^ or Hg^2+^ were tested per cell. The volume of the chamber contained 1 mL of extracellular solution. Thus a maximum of 20 µL of stock solution was added to the extracellular solution during experiment.

The concentration of Zn^2+^ producing 50% of the maximal effect (EC50) was 7.9±1.2 µM for the control VSMCs and 7.4±1.4 µM for the VSMCs pretreated with 6-Bnz-cAMP. The Hill coefficient of the fitted curve was 2.2±0.1 for the control and 1.2±0.2 for the 6-Bnz-cAMP pretreated VSMCs. The concentration of Hg^2+^ producing 50% of the maximal effect (EC50) was 7.6±1.2 µM for the control VSMCs and 6.9±1.5 µM for the VSMCs pretreated with 6-Bnz-cAMP. The Hill coefficient of the fitted curve was 2.3±0.3 for the control and 1.1±0.2 for 6-Bnz-cAMP pretreated VSMCs.

## Discussion

The important findings of our study are: 1) cAMP agonists did not inhibit K2P currents activated by PKC; 2) however, if VSMCs were pretreated with cAMP agonists before PKC activation than they inhibited large fraction of the PDBu-induced K2P currents; 3) cAMP agonists inhibited K2P currents without PKA activation; 3) K2P currents inhibited by cAMP agonists were affected by divalent cations and alkaline pH. Also, PKA blockers did not prevent the inhibitory effect of the intracellular cAMP on PDBu-elicited K2P currents. These results indicate that 6-Bnz-cAMP and other cAMP analogs may inhibit PDBu-elicited K2P currents through a PKA-independent mechanism. cAMP analogs may interact with other unidentified proteins involved in K2P channel regulation in VSMC.

In other vascular preparations it was shown that acetylcholine binds to and activates muscarinic receptors in endothelial cells and TREK-1 is inhibited by PKC phosphorylation. In response to the subsequent depolarization of endothelial cells, NO was generated, and it diffused to the neighboring VSMCs and relaxed them [50]. The vascular reaction to acetylcholine was also impaired in TREK-1−/− mice, indicating the importance of TREK-1 in the process of endothelium dependent vasodilatation [51].

However, these mechanisms are not general in all regions of the cerebral circulation. In our preparation, PKC agonist PDBu produced strong activation of outward K^+^ currents. Differences between cellular mechanisms regulating vessel diameter of the aorta and other blood vessels are closely related to the unique structure and function of the aorta [39], [40], [41]. The aorta should simultaneously damp the amplitude of pressure pulses (Windkessel mechanism) and provide volume by vasodilation needed to accommodate reflected central waves when resistance vessels contract [52]. This hypothesis may explain why phosphorylation of K^+^ channels by PKC in the aorta increases their open probability in contrast to other blood vessels. Protein kinase C have been found to not only inhibit K2P channels but also to inhibit delay rectifier and Ca^2+^-dependent K^+^ currents in VSMCs in small muscular arteries [36], [37], [38], [53]. Whereas, in our preparation, PKC activated by PDBu (1 µM) increased the amplitude of the K2P currents and of other outward K^+^ currents that are inhibited by conventional K^+^ channel blockers.

The question as to whether PKC is indispensable for the regulation of K2P channels open probability remains under debate. Stimulation of Gq-coupled receptors was found to inhibit TREK activity by a mechanism involving protein kinase PKC and channel phosphorylation [54], [21], [15]. Different (NH2-terminal) splice variants of TREK-2 were also inhibited by PMA similarly to TREK-1, indicating that the PKC-mediated regulation of TREK-2 was also operational [55]. Robust pharmacological activation of PKC by the phorbol ester PMA inhibited TASK-3 channel [14], [56]. However, the receptor-mediated inhibition of the channel was not influenced by pharmacological inhibition of PKC [54]. In our preparation, pretreatment of the cells with Gö 6976 strongly inhibited PDBu-elicited increase of the amplitude of K2P currents. Gö 6976 has been reported to inhibit the Ca^2+^-dependent isozymes alpha and beta, whereas even a micromolar concentration of Gö 6976 had no effect on the kinase activity of the Ca^2+^-independent PKC subtypes delta, epsilon, and zeta [57], suggesting that these types of PKC isozymes affect K2P currents in the VSMCs isolated from the aorta.

The failure of specific PKA antagonists (H-89, Rp-cAMPS) to alter the inhibition of K2P currents suggests that cAMP can inhibit K2P currents through a PKA independent mechanism. This hypothesis is supported by an earlier report that suggested that cAMP might inhibit the TREK1 channel independently from PKA in bovine adrenal zona fasciculata cells [27]. However, our experiments do not completely exclude the involvement of PKA. Interestingly, cAMP analogs have been reported to enhance TREK1 mRNA independently from PKA in adrenocortical cells [58]. Finally, cAMP was also shown to have other effects independent of PKA, suggesting that other targets of cAMP may modulate activity of K2P channels [59], [60], [61], [62], [63].

Exchange proteins directly activated by cyclic AMP (Epacs or cAMP-GEF) represent a family of novel cAMP-binding effector proteins. Epacs are the new target of cAMP and present an alternative to PKA cellular mechanism regulating ion channel activity in vascular tissue. Recently it was elegantly demonstrated that Epac exists in a complex with vascular ATP-sensitive potassium (KATP) channel subunits and that cAMP-mediated activation of Epac modulates K_ATP_ channel activity in rat aortic smooth muscle cells [64]. However, elevation of intracellular cAMP in vascular smooth muscle is associated with activation of PKA, which phosphorylates the K^+^ channels, that leads ultimately to vasorelaxation. This raises the question as to under what conditions a cAMP-mediated Epac-dependent inhibition of vascular K+ channel activity would occur? Dart and coauthors [64] proposed an original hypothesis that Epac activation acts as a feedback regulator of K^+^ channel function following large fluctuations of cAMP. Their hypothesis is based on the observation that while in vitro cAMP affinity between Epac and PKA are virtually identical, the concentration of cAMP required for half-maximal activation of Epac1 is reported to be higher compared to the required concentration for PKA. The implication is that PKA may be preferentially activated by small elevations in cAMP. This mechanism might work for the regulation of K2P channels activated by ATP via metabotropic purinergic receptors in vascular smooth muscle cells isolated from mouse aorta. We have earlier reported that ATP recruits both adenylate cyclase (AC) and phospholipase C (PLC) pathways in myocytes isolated from the aorta, providing a physiological basis for the Epac and PKC to regulate K2P channels activity [24], [25], [28].

The concept of cell diversity that describes the vascular medium as a mosaic of functionally and morphologically different cell types is now generally accepted [65], [66], [67]. Vascular smooth muscle cell types may vary from segment to segment within an artery and may contrast between vascular trees of different organs. Our findings are in agreement with this concept. Three types of effects evoked by divalent cations on PDBu-elicited K2P currents can be explained by the binding of mercury and zinc to TREK, TRESK, TASK and TWIK channels. Biphasic responses could be generated by simultaneous binding of divalents to K2P channels with different binding affinities. Zn^2+^ and Hg^2+^ have been suggested to have binding sites on the extracellular side of K2P channels [47], [48], [13], [66]. Because cAMP analogs inhibited increase of the PDBu-elicited K2P currents produced by divalent cataions, we suggest that cAMP may inhibit TREK1 channel without PKA activation. The shift of the sensitivity of K2P currents in cells pretreated with cAMP analogs to acid pH supports this hypothesis. The unequal expression of K2P channels may create smooth muscle cells with different resting membrane potentials and thereby regulate intracellular calcium responses and propagation of calcium waves between smooth muscle cells. It should be noted that TREK channels are sensitive to mechanical strain of the membrane and their unequal expression may also regulate local stretch elicited responses induced by pulsatile blood pressure and tune rebound of the aorta.

K2P channels in the aorta may have therapeutic potential. Aortic dysfunction, such as arterial stiffness that occurs with increasing age, is an intense area of research under the general heading of “vascular stiffness” [68], [69], [70]. Selective manipulation of the open probability of K2P channels in the aorta may help to decrease pulse pressure and prevent microvascular damage.
